# Quick remedy commits and their impact on mining software repositories

**DOI:** 10.1007/s10664-021-10051-z

**Published:** 2021-10-28

**Authors:** Fengcai Wen, Csaba Nagy, Michele Lanza, Gabriele Bavota

**Affiliations:** Software Institute, USI Università della Svizzera italiana, Lugano, Switzerland

**Keywords:** Fixing commits, Empirical software engineering, Mining software repositories

## Abstract

Most changes during software maintenance and evolution are not atomic changes, but rather the result of several related changes affecting different parts of the code. It may happen that developers omit needed changes, thus leaving a task partially unfinished, introducing technical debt or injecting bugs. We present a study investigating “*quick remedy commits*” performed by developers to implement changes omitted in previous commits. With *quick remedy commits* we refer to commits that (i) *quickly* follow a commit performed by the same developer, and (ii) aim at *remedying* issues introduced as the result of code changes omitted in the previous commit (e.g., fix references to code components that have been broken as a consequence of a rename refactoring) or simply improve the previously committed change (e.g., improve the name of a newly introduced variable). Through a manual analysis of 500 quick remedy commits, we define a taxonomy categorizing the types of changes that developers tend to omit. The taxonomy can (i) guide the development of tools aimed at detecting omitted changes and (ii) help researchers in identifying corner cases that must be properly handled. For example, one of the categories in our taxonomy groups the *reverted commits*, meaning changes that are undone in a subsequent commit. We show that not accounting for such commits when mining software repositories can undermine one’s findings. In particular, our results show that considering completely reverted commits when mining software repositories accounts, on average, for 0.07 and 0.27 noisy data points when dealing with two typical MSR data collection tasks (i.e., bug-fixing commits identification and refactoring operations mining, respectively).

## Introduction

In the software life-cycle, change is the rule rather than the exception. Changes are generally performed through commit activities to add new functionality, repair faults, and refactor code (Mockus and Votta 2000). Some of these commits can involve a substantial part of the source code, with dozens of artifacts impacted (Hattori and Lanza 2008). This is often the result of what Herzig and Zeller (2013) defined as *tangled commits*: Commits grouping together several unrelated activities, such as fixing a bug and adding a new feature.

In other cases, a single cohesive change (e.g., a bug fix) is instead split across several commits. This can be due to omitted code changes and/or the need for fixing a mistake done in the first attempt to implement the change. Park et al. (2012) showed that 22% to 33% of bugs require more than one fix attempt (i.e., supplementary patches). Studying supplementary patches can be instrumental in designing recommender systems able to reduce omission errors by alerting software developers, as attempted in a subsequent work by Park et al. (2017), where the authors tried to predict additional change locations for real-world omission errors. Due to the limited empirical evidence about the nature of omitted changes, this is still an open challenge. Indeed, while the work by Park et al. (2012) investigates omitted changes, it explicitly focuses on supplementary patches for bug-fixing activities, ignoring other types of code changes (e.g., implementation of new features, refactoring). Thus, there is no study broadly investigating the types of changes that developers tend to omit during implementation activities.

To fill this gap, in our previous work (Wen et al. 2020) we presented a qualitative study focusing on “*quick remedy commits*” performed by developers. We defined as *quick remedy commits* those commits that (i) quickly succeed a commit performed by the same developer in the same repository; and (ii) aim at remedying the issues introduced as the result of code changes omitted in the previous commit (e.g., fix references to code components that have been broken as a consequence of a rename refactoring) and/or improves suboptimal choices made in the previously committed change (e.g., refactoring code to improve its comprehensibility). In other words, we identified pairs of commits (*c*_*i*_, *c*_*i*+ 1_) that are temporally close (i.e., *c*_*i*+ 1_ succeeds *c*_*i*_ by a few minutes), are performed by the same developer, and include in the commit note of *c*_*i*+ 1_ a reference to fixing issues introduced in *c*_*i*_.

**Fig. 1:**
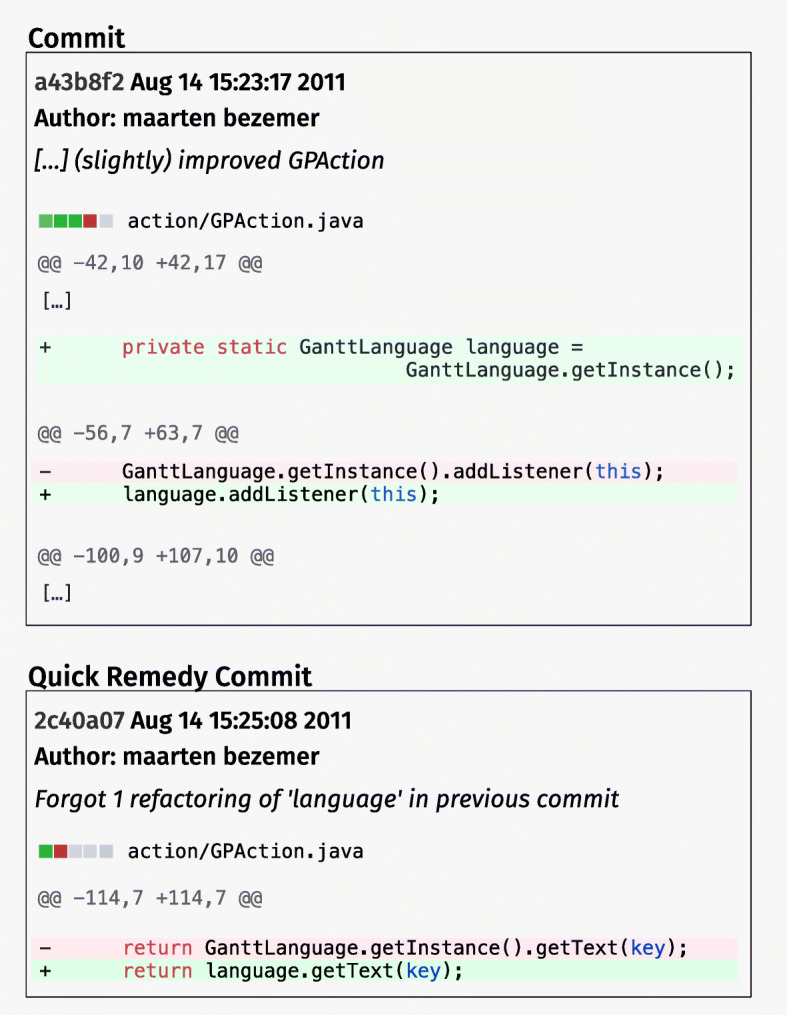
Example of quick remedy commit

Figure 1 shows an example of a quick remedy commit from our dataset, and in particular from the GitHub project . In the commit depicted in the top part of Fig. 1 (i.e., commit ), the developer implemented, among other changes, a refactoring aimed at simplifying the code of the class. In particular, instead of invoking three times the method in different parts of the class, the variable is instantiated, and reused where needed.

Two minutes later, the same author performs a *quick remedy commit* (bottom part of Fig. 1 — commit ) by reporting in the commit note: *Forgot 1 refactoring of ’language’ in previous commit*. The remedy commit propagates the changes introduced by the refactoring to another location of the class, that was missed by mistake in the original commit.

We decided to focus on remedy commits (*c*_*i*+ 1_) that are temporally close to the original change they fix (*c*_*i*_) for two reasons. First, it is easier to establish a clear link between two commits by the same developer if they are performed within a few minutes. Second, as shown by Park et al. (2017), it is challenging to prevent omission errors automatically; thus, we decided to focus on omission errors that, since fixed within few minutes, are likely not to be so complex.

This allows gathering empirical knowledge to take a first step in automating the prevention of a basic set of omission errors that, as we show, can be responsible for bugs and major code inconsistencies if not promptly fixed.

We defined heuristics to identify *quick remedy commits* automatically, and mined the commits of interest from the complete change history of 1,497 Java projects hosted on GitHub. This allowed the identification of $\sim $1,500 candidates quick remedy commits. We manually analyzed 500 of them looking at the changes introduced in the remedy commit (*c*_*i*+ 1_) and the previous commit (*c*_*i*_) as well as the summary of changes provided in the commit notes.

The goal of the manual analysis was to identify the rationale of the remedy commits to define a taxonomy categorizing the types of issues introduced by developers during commit activities that trigger a remedy commit, discussing the implications of our taxonomy for researchers and practitioners.

In this work, extending our previous paper (Wen et al. 2020), we further looked into the implications of a specific part of our taxonomy for researchers working in the Mining Software Repositories (MSR) field. In particular, we focused on a category in our taxonomy grouping together *reverted commits*, i.e., remedy commits *c*_*i*+ 1_ in which the developers revert, completely or partially, the code changes they committed in the previous commit *c*_*i*_. We defined a methodology to automatically identify these commits in a given repository and studied the impact they could have on MSR studies. The decision to focus on such a specific category in our taxonomy is two-fold: (i) as we will explain later in the paper, it is the type of quick remedy commits that is more likely to affect the data collection process in MSR, possibly leading to the inclusion of noisy data points in the study; (ii) it is the only category for which a reliable and automated detection mechanism can be easily devised (i.e., it is relatively easy to detect reverted commits as compared to other categories of commits in our taxonomy).

We took two “data collection tasks” frequently performed in MSR studies, namely the identification of bug-fixing commits (see e.g., Rodriguez-Perez et al. 2017b; Rodríguez-Pérez et al. 2018; Tufano et al. 2018; Wang et al. 2020; Penta et al.^2020^) and the mining of refactoring operations performed in the history of a system (see e.g., Penta et al. 2020; Peruma 2019; Mahmoudi et al. 2019; Lin et al. 2019; Fakhoury et al. 2019; AlOmar et al. 2019). Then, we applied these two tasks on 100 long-lived Github repositories; collecting refactoring operations performed in each commit and a set of bug-fixing commits. Finally, we cleaned the collected data by removing completely and partially reverted commits. For example, a researcher may identify a bug-fixing commit in the history of a software system. However, if such a bug-fix is later on reverted by the developer, we argue that, in most of the cases, it should not be considered as a valid data point, since it basically represents noise. We show that, for each completely reverted commit kept in the collected data, there is a .07 increase in the number of detected bug-fixing commits and a 0.27 increase in the number of detected refactoring commits. The methodology we adopt to identify the *reverted commits* can be applied in MSR studies to help researchers in minimizing the impact of these commits on their findings. Clearly, the removal of *reverted commits* is subject to the goal of the study and the data analyses researchers are interested in performing. For example, if the goal of the study is to count the number of bugs introduced by a developer in a system, researchers may be willing to also count bug-introducing commits that have been later on reverted. Instead, if the goal is to assess the logical coupling between code components (i.e., how frequently they co-change), researchers may want to ignore completely reverted changes in the coupling computation. Our study confirms the importance of careful data cleaning when mining software repositories, as highlighted in previous works (Rigby and Robillard 2013).

The data used in both our studies are publicly available (Replication package 2021).

### Structure of the paper

In Section 2 we present the design and the results of our first empirical study, in which we investigate the types of quick remedy commits performed by developers. Section 3 presents the design and results of our second study, assessing the potential impact of reverted commits in MSR studies. In Section 4 we discuss the threats that could affect the validity of our two studies, while in Section 5 we discuss the related literature. In Section 6 we conclude the paper and outline our future work.

## Study I: Studying Quick Remedy Commits Performed by Developers

### Study Design

The *goal* of the study is to qualitatively investigate quick remedy commits. The *purpose* is to define a taxonomy of quick remedy commits that developers perform to fix issues introduced in a previous commit and/or finalize an uncompleted implementation task. The study addresses the following research question (RQ):

**RQ**_**1**_: *What types of quick remedy commits are made by developers in Java projects?*

This RQ aims at identifying the types of quick remedy commits that are performed by developers (e.g., documenting through a code comment a piece of code introduced in the previous commit). Knowing the types of quick remedy commits made by developers can guide the development of tools to automatically alert developers when code changes they are committing may require a subsequent remedy commit. In some cases this could even avoid the introduction of bugs (e.g., due to changes not propagated in all code areas where they are required).

#### Data Collection and Analysis

To answer RQ_1_ we mined the complete change history of 1,497 open source Java projects hosted on GitHub. These projects represent the context of our study and have been selected from GitHub in November 2018 using the following constraints:

- **Programming language.** We only considered projects written in Java since all the manual evaluators involved in the study (i.e., three of the four authors) have experience in Java, and would be able to understand the reasons behind the quick remedy comments in most of the cases.
- **Change history.** Since we were interested in identifying a good number of quick remedy commits to manually analyze, we only selected projects having a relatively long change history, composed of at least 500 commits.
- **Popularity.** The number of stars (About stars (GitHub) 2021) of a repository is a proxy for its popularity on GitHub. Starring a repository allows GitHub users to express their appreciation for the project. Projects with less than ten stars are excluded from the dataset, to avoid the inclusion of likely irrelevant/toy projects.

A total of 6,563 projects satisfied these constraints. Then, we sorted the projects in descending order based on their number of stars (i.e., the most popular on top), and we manually inspected the ranked list (starting from the top) to filter out repositories that do not represent real software systems (e.g., java-design-patterns 2021 and spring-petclinic 2021). Such a selection was done by checking the projects’ names and descriptions (no code analysis was performed). We also checked for projects with shared history (i.e., forked projects). In particular, we considered as forked projects two repositories having in their history at least one commit having the same *SHA* and commit date. When we identified a set of forked projects, we only selected among them the one with the longest commit history (e.g., both FindBugs 2021 and its successor SpotBugs 2021 fall under our search criteria, but we only kept the latter one). Such a process stopped once we reached 1,500 valid projects for our study.

During the cloning of the 1,500 GitHub repositories, we got a cloning error for three of them. Thus, we extracted the list of commits performed over the change history of the remaining 1,497 projects. Table 1 reports descriptive statistics for size, change history, and popularity of the selected projects. The complete list of considered projects is publicly available in our replication package (2021).

**Table 1:** Dataset statistics

	Overall	Per Project	
		Mean	Median
Java files	1,599,323	1,068	360
Effective LOC	162,243,714	108,379	31,392
Stars	2,895,219	1,930	762
Commits	7,926,912	5,313	1,778

To extract the history of the subject systems, we iterated through the commit history related to all branches of each project with the --- command. This allowed us to analyze all branches of a project, without intermixing their history and avoiding unwanted effects of merge commits.

Then, given the commit history, our goal was to identify all pairs of subsequent commits (*c*_*i*_,*c*_*i*+ 1_) in which *c*_*i*+ 1_ had been performed by a developer *D*_*j*_ as a quick remedy fix for a commit *c*_*i*_ also authored by *D*_*j*_. In other words, *c*_*i*+ 1_ must (i) have been authored by the same developer of *c*_*i*_ and performed within a relatively short time interval from *c*_*i*_; (ii) clearly be a “compensatory” fix for *c*_*i*_.

**Fig. 2:**
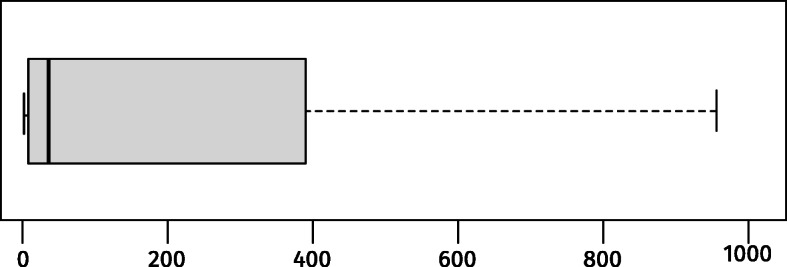
Time differences (in minutes) between subsequent commits (without outliers)

To identify the (*c*_*i*_,*c*_*i*+ 1_) pairs of interest, we adopt the following heuristic-based procedure. First, we computed the time interval between all adjacent (subsequent) commits in each system authored by the same developer. In *git* it is possible to retrieve the *author date* (i.e., the date in which the change has been implemented by the author) or the *committer date* (i.e., the date in which the change has been committed). Given the goal of our work, we considered the *author date*. We analyzed the distribution of these time intervals (see Fig. 2).

We considered the first quartile, exactly *five minutes*, as a candidate threshold to identify remedy commits: *c*_*i*+ 1_ commits performed as quick fixes for their predecessor *c*_*i*_ commit. This allowed us to select pairs of commits meeting our first requirement: They were authored by the same developer and performed in rapid succession (i.e., within five minutes). This filtering left us with 1,041,397candidate commits.

Second, we set up a process to define lexical patterns allowing the identification of *c*_*i*+ 1_ commits in which the developer explicitly indicates in the commit notes the fact that *c*_*i*+ 1_ is a remedy commit for changes introduced in the previous commit (*c*_*i*_). The first author extracted from all 1,041,397commits output of the previous filtering step the words and 2-grams used in their commit notes. This means that, from a commit note reporting “*Fixes a bug introduced in previous commit*”, we would extract *fixes*, *a*, *bug*, etc. as the single words, and *fixes a*, *a bug*, *bug introduced*, etc. as 2-grams. To remove noise, stop words (e.g., articles) and all single words shorter than four characters had been excluded from the set of single words (not from the 2-grams list). The remaining words and all 2-grams had then been sorted by frequency in descending order, excluding the long tail of those appearing in less than ten commits. Indeed, even if useful to identify remedy commits, lexical patterns defined from these words/2-grams are unlikely to retrieve a substantial amount of useful commits and, thus, are excluded *a priori* from reducing the inspection effort. For each remaining word/2-gram, we randomly extracted ten commit notes in which it appears.

This dataset, composed of words/2-grams and related commit notes, had been manually and independently inspected by three authors with the goal of defining the needed lexical patterns. After an open discussion in which each author presented his list of patterns, the three evaluators agreed on the following lexical pattern to identify remedy commits:

(*former* or *last* or *prev* or *previous*) and *commit*

This means that commit notes including *former commit*, *last commit*, *prev commit*, or *previous commit* would be matched and considered as relevant for our study. While this heuristic is quite strict, our goal was to maximize precision at the expense of recall, considering the fact that our study is qualitative in nature and does not target a large number of manually analyzed commits. At the end of this last filtering step, we obtained 1,577*c*_*i*+ 1_ commits which (i) have been authored within five minutes from the commit *c*_*i*_ previously performed by the same author; and (ii) explicitly mention in the commit note a lexical reference to the previous commit that can be captured by the defined pattern. Given the high cost of the manual analysis process detailed in the following, we decided to focus our analysis on a randomly selected sample of 500 commits, representing a 99% statistically significant sample with a 4.8% confidence interval.

The 500 commits were randomly distributed among three authors, making sure that each commit was classified by two authors. The goal of the process was to identify the exact reason behind the changes performed in the commit. If the commit was unrelated to the previous one, the evaluator classified it as *false positive*.

Otherwise, a tag explaining the reason for the change (e.g., *remove debugging code from the previous commit*) was assigned.

**Fig. 3:**
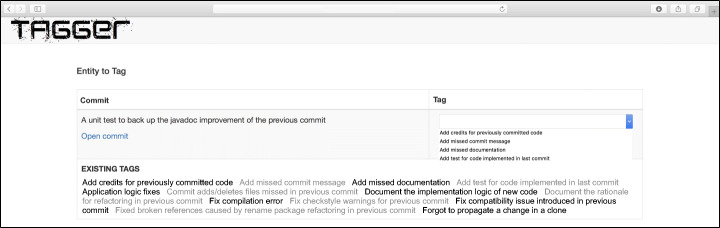
Web application used to run the manual tagging

We did not limit our analysis to the reading of the commit message, but we analyzed the source code diff of the changes implemented in the GitHub commits, both in the *c*_*i*+ 1_ commit as well as in its predecessor (*c*_*i*_). The tagging process was supported by a Web application that we developed to classify the commit and to solve conflicts between the authors. The Web application is shown in Fig. 3. Each author independently tagged the commits assigned to him by defining a tag describing the reason behind the commit. Every time the authors had to tag a commit, the Web application also showed the list of tags created so far, allowing the tagger to select one of the already defined tags (visible in the bottom part of Fig. 3). Although, in principle, this is against the notion of open coding, in a context like the one encountered in this work, where the number of possible tags (i.e., cause behind the commit) is extremely high, such a choice helps using consistent naming and does not introduce substantial bias. In cases for which there was no agreement between the two evaluators (44%of the classified commits), the commit was assigned to an additional evaluator to solve the conflict. While such a percentage may look high, it is worth considering that our task was not to assign commits to a list of predefined categories, but to define the names for such categories during the tagging process. This naturally leads to a higher number of conflicts. Also, we considered as a conflict cases in which a different but “semantically equivalent” tag was used by the two evaluators (e.g., *remove unnecessary code* *vs* *remove unneeded code*). In this case, the third evaluator just made sure that consistent wording was used, and selected the proper tag. In a minority of cases, the two evaluators applied completely different tags and the third evaluator could choose whether to reuse one of the two labels or, instead, define a new tag by discussing and agreeing with the two original evaluators.

**Fig. 4:**
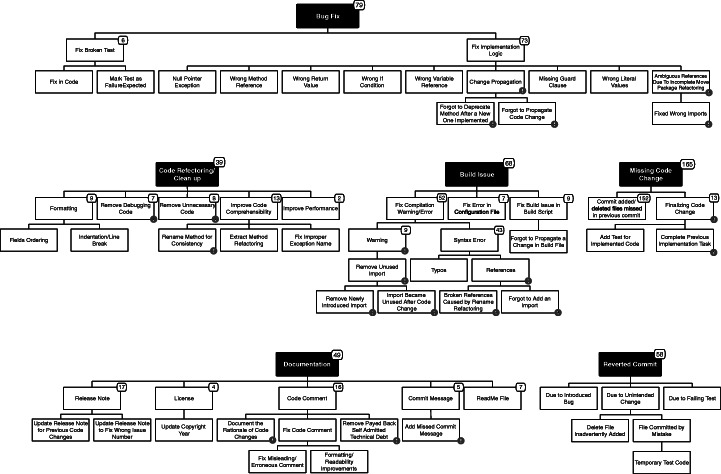
Taxonomy of quick remedy commits

After having manually tagged all commits, we defined a taxonomy of quick remedy commits through an open discussion involving all the authors (see Fig. 4). We qualitatively answer our research question by discussing specific categories of commits likely related to the code changes developers often forget to implement and try to immediately remedy. For each category, we present interesting examples and discuss implications for researchers and practitioners.

### Results

We addressed our research question by labeling 500 commits identified as candidates to being quick remedy commits (see Section 2.1). We identified 42 false positives (i.e., commits *c*_*i*+ 1_ that were not related to the preceding *c*_*i*_ commit) and 458 commits actually classifiable as quick remedies.^1^ Note that not all these quick remedy commits are compensatory fixes for issues caused by omitted changes. They also include fixes for previously introduced errors (e.g., the developer realizes that her previous commit introduced a bug) as well as commits aimed at simply improving the previously committed change (e.g., improve the name of a newly introduced variable). Finally, our taxonomy also features remedy commits aimed at fixing simple mistakes performed during the *c*_*i*_ commit process itself (e.g., the developer forgot to include a modified file in commit *c*_*i*_ and thus commits it in *c*_*i*+ 1_).

Overall, we identified 69 types of quick remedy commits made by developers, 20 of which relevant for changes omitted in the previous commit.

Figure 4 presents the results in the form of a hierarchical taxonomy composed by six root categories: *Bug Fix*, *Code Refactoring/Clean Up*, *Build Issue*, *Missing Code Change*, *Documentation*, and *Reverted Commit*. The more specific types of quick remedy commits are represented either as intermediate nodes or leaves, and commits relevant for the fixing of issues caused by omitted changes are marked with a  sign. For each category, we next describe representative examples and discuss implications for researchers (indicated with the  icon) and/or practitioners ( icon) derived from our findings.

#### Bug Fix (79)

This category groups pairs of commits (*c*_*i*_, *c*_*i*+ 1_) in which the remedy commit (i.e., *c*_*i*+ 1_) fixes a bug introduced in *c*_*i*_. We identified two main subcategories: *Fix Broken Test*, in which *c*_*i*+ 1_ has been triggered by test cases failing after the change implemented in *c*_*i*_, and *Fix Implementation Logic*, in which the developer realized that she introduced a bug in *c*_*i*_ and quickly submits a patch.

The commits in the *Fix Broken Test* category targets the fixing of the production code or the test code modified in *c*_*i*_ that caused a break in the test suite. For example, in the project of , a developer reported in the commit message: “*Fix tests broken by former commits*” (Commit to denominator project on GitHub 2021).

While in the cases we analyzed the issue was spotted and fixed quickly by the developer, there might be non-trivial cases in which only a subset of the test suite is executed for regression testing (e.g., due to a limited testing budget) and a non-executed broken test is not identified by the developer.

 For researchers, this is an opportunity to study test breaking-changes and to develop techniques able to alert the developer when a change she implemented might require a double check of (part of) the test suite.  For practitioners, continuous integration practices can help in timely spotting these issues in most of the cases.

The fixes to the implementation logic are mostly classic bugs introduced but quickly recognized and fixed by developers (e.g., errors in conditions, wrong literal values, null pointer exceptions, etc.). While these are not related to omitted changes, they are interesting since they represent bugs fixed by developers within five minutes (due to our selection criteria for the commits).

This indicates that these bugs, while prevalent in our taxonomy (73 instances), are likely quite simple to fix. Thus,  researchers could investigate the possibility of creating approaches able to learn from this data on how to avoid and/or automatically fix these bugs. For example, recent work applied Neural Machine Translation (NMT) models to automatically fix bugs (Tufano et al. 2018). However, given the complexity of this task and the non-trivial bugs that these models have to fix, they are usually only able to automatically fix a minority of the bugs provided as input (Tufano et al. 2018). Focusing on these simpler but quite frequent bugs could represent a good application scenario for the NMT-based bug fixing approach.

Some of the fixes in the *Fix Implementation Logic* category are related to omitted changes (see Fig. 4). This includes the *Forgot to Propagate Code Change* category in which developers do not consistently propagate a change across all relevant code components. This is typical of cases in which code clones are spread in the system and inconsistent changes are implemented in *c*_*i*_ (Krinke 2007). An example of this can be seen in the *mathttTomP*2*P* project. In a commit (Commit to TomP2P project on GitHub 2021b), the developers adapts a builder class () to earlier changes of the original class and they implement new methods such as and . In a follow-up change (Commit to TomP2P project on GitHub 2021c), they fix a conditional statement to check the status of a object in a new branch. Then, only a few seconds later (Commit to tomp2p project on GitHub 2021a), they update a conditional check with a similar structure but in another class. For this last commit, the commit message says “*belongs to previous commit*”. Another example can be seen in the *mathttspacewalk* project. In a commit (Commit to spacewalk project on GitHub 2021a), they update a SQL script by adding a query for the removal of unnecessary data. Then, in the quick subsequent commit (Commit to spacewalk project on GitHub 2021b), they propagate the same schema changes into a database upgrade file.

 These examples highlight the relevance for practitioners of approaches to guide code changes (see e.g., the seminal work in the area by Zimmermann et al. (2005)) as well as the need for  the research community to continue improving these techniques and, possibly, making them easily pluggable into a continuous integration pipeline to foster developers’ adoption.

Interesting in this category is also the introduction of *ambiguous references due to incomplete move package refactoring*. We found this case in the project, where they migrate some classes to another package (Commit to Accumulo project on GitHub 2021), but still keep the old ones.

In a follow-up commit (Commit to accumulo project on GitHub 2021), they realize that they use, however, the wrong references to the migrated classes.  Code clone detection techniques (Roy et al. 2009) could help in these cases by promptly pointing the developer to the presence of multiple copies of the same classes in the repository. The integration of these approaches in a just-in-time fashion could help in identifying clones introduced in the last commit, thus avoiding mistakes as the one in the discussed commit (Commit to Accumulo project on GitHub 2021).

#### Code Refactoring/Clean up (39)

This category groups the pairs of commits (*c*_*i*_, *c*_*i*+ 1_) in which the remedy commit (i.e., *c*_*i*+ 1_) implements a refactoring/cleanup of the code changed in *c*_*i*_ (see Fig. 4). In these commits developers are either not satisfied of the code they implemented or are trying to address warnings received by static analyzers.

Some other subcategories include the simple removal of code that was only temporary implemented in *c*_*i*_ (i.e., *Remove Debugging Code*) or that becomes unnecessary after *c*_*i*_’s changes (i.e., *Remove Unnecessary Code*). Also, code formatting issues (e.g., mainly the inconsistencies of indentations and line breaks introduced with code changes) were fixed by developers in the remedy commit (ie *Code Formatting*). Additionally, in 2 cases, developers changed the code implemented in *c*_*i*_ to improve its performance. An example can be seen in project (Commit to lombok project on GitHub 2021) where a developer fine tunes a cache clearing mechanism implemented in a previous commit by turning a variable volatile and moving the invocation for the cache clearing after a conditional check.

However, the main purpose of those code refactoring/clean up tasks is to improve the code understandability. Variable and method renaming refactoring (i.e., renaming a variable or method to better reflect its functionality) is the most common way to make the code easier to comprehend. Also popular are code transformations aimed at replacing literal values with variables or splitting long functions through extract method refactoring. The latter allows not only to foster comprehensibility, but also the reusability of small code snippets.

Other interesting cases are the ones in which developers modify the previously committed code to promote consistency with the coding style of the project (see e.g., *Rename Method for Consistency*). For example, in a commit of the project (Commit to liferay-portal project on GitHub 2021), developers opened an issue to “*introduce tests to document current behavior*” (Liferay Portal Issue LPS-44476 2021). Interestingly, in this process they very carefully review the used method names for better readability, and in a commit they say:

*[...] where specific method names are NOT accurate, go for a generic name to force the developer to read the code to find what the method actually does*.

The developers decided to change a method’s name from to . In the next commit (Commit to liferay-portal project on GitHub 2021), to remain consistent, they replace the method invocation of (in another class) to . For this last commit, the commit message says “*Match previous commit even though this method name was accurate*”.

 The inconsistencies fixed with simple refactorings point to the possibility for the software engineering research community to investigate techniques able to learn coding conventions used in a given system and recommend fixes for possible violations. To the best of our knowledge, the only attempt at date has been made by Allamanis et al. (2014) with their NATURALIZE tool able to recommend meaningful identifier names and formatting guidelines. Other approaches focus only on rename refactoring suggestions (Lin et al. 2017, 2017). While these techniques cover most of the inconsistencies fixed in the *Code Refactoring/Clean up* category (e.g., *Rename Method for Consistency*, *Fix Improper Exception Name*), others are left uncovered (e.g., *Fields Ordering*), indicating more potential for additional research in the area of recommending coding convention fixes.

#### Build Issue (68)

This category is related to commits fixing build issues introduced as a result of the *c*_*i*_ changes. The main subcategory here is the fix of the compilation errors/warnings issued by the compiler due to the changes in *c*_*i*_ (i.e., *Fix Compilation Warning/Error*). Unused import statements are the main cause for the warnings we identified (see Fig. 4), and the trigger for the remedy commits in this category. The unnecessary import statements are caused either by statements introduced in *c*_*i*_ by the developer and then unused, or by previously existing becoming unused due to the changes implemented in *c*_*i*_. These warnings are usually raised by static analysis checks performed at commit time and, thus, are easy to catch for developers.

In the *Syntax Error* category we found many cases of broken references due to rename refactoring operations performed in *c*_*i*_. These rename refactorings are related to variables, methods, classes, as well as packages. An example can be seen in the commit (Commit to tower project on GitHub 2021) of the project which followed a renaming of multiple classes. Some other cases were violating the syntax of the programming language due to introduced typos (e.g., missing statement separators).

Considering the good refactoring support provided by modern IDEs, the identification of these broken references as a consequence of refactorings was quite surprising for us.  This may indicate either that these refactorings were performed manually, leading to the introduction of broken references, or that bugs might affect refactoring engines, as already found by previous work in the literature (Daniel et al. 2007). Additional investigation focused on these specific types of errors is needed to understand the reasons behind them.

Other subcategories that also caused a build issue include the fix of introduced errors in configuration files (i.e., *Fix Error in Configuration File*) or in a build script (i.e., *Fix Build Issue in Build Script*). For example, in some remedy commits developers fixed broken tags in configuration files or incorrect filepath references in build scripts.

#### Missing Code Change (165)

This category groups the pairs of commits (*c*_*i*_, *c*_*i*+ 1_) in which the remedy commit (i.e., *c*_*i*+ 1_) adds some missing code changes that should be introduced within previous commit *c*_*i*_. We divided those commits into two subcategories: *Commit Added/Deleted Files Missed in Previous Commit* and *Finalizing Code Change*.

The first subcategory is related to fixing a previous commit error. In this case, we are not referring to the code changes implemented in *c*_*i*_, but to the commit process itself. This issue is mainly caused by an incorrect selection of committed files by the developer. Also, sometimes IDE cache issues can lead to a similar situation (e.g., the IDE cached the wrong version of a committed file or lost track of some code changes during the git commit process). While this subcategory is kind of unrelated to artifacts’ changes, it still provides hints for interesting research directions.  For example, approaches to automatically identify the set of files to commit can be designed to reduce the possibility of missing files or to include unrelated changes. This could also go further and recommend to the developer *when* to commit in such a way to avoid tangled commits (Herzig and Zeller 2013) and committing cohesive sets of code changes. To the best of our knowledge, the only step in this direction has been done by Bradley et al. (2018) with a context-aware developer assistant able to identify the files to push towards the repository when the developer asks. However, more automation can be envisioned, with approaches also able to (i) recommend when to commit (as previously said, to e.g., avoid tangled commits), and (ii) summarize the changes in a meaningful commit message (as attempted by Jiang et al. 2017).

The second subcategory (i.e., *Finalizing Code Change*) refers to code changes forgotten or left incomplete for other reasons in commit *c*_*i*_ that are then finalized in *c*_*i*+ 1_. This includes cases in which developers add new test cases needed to test the production code introduced in the previous commit, or to complete an implementation task. For example, in a commit of the project (Commit to openpnp project on GitHub 2021), the developer claimed in the commit message that three new sub-features were introduced. However, the developer forgot to actually implement one of those sub-features and added the missing implementation in the following commit. In another case from the project (Commit to geoserver project on GitHub1 2021), the developer introduced a guard clause in commit *c*_*i*_ to check if a processed reference is . Meanwhile, a debugging message was also added saying that “*the reference is null, reset it to default value*”. However, the actual implementation for resetting this reference value was missing in commit *c*_*i*_, and implemented in the remedy commit *c*_*i*+ 1_.  While these issues are of different natures, some of them can be spotted automatically through techniques comparing what is described in the commit message and what has been actually implemented in the change. For example, in the previously discussed example (Commit to openpnp project on GitHub 2021), a misalignment between the number of sub-features actually implemented and claimed in the commit message could be spotted and reported to the developer.

#### Reverted Commit (58)

This category groups remedy commits *c*_*i*+ 1_ in which the developers revert the code changes they committed in the previous commit *c*_*i*_. The reasons pushing a developer to revert previous changes through a remedy commit include: (i) introduced bugs spotted after pushing the changes in *c*_*i*_; (ii) unintended changes, pushed in *c*_*i*_ by mistake; (iii) failing test cases, possibly indicating a bug worth of investigation before applying the *c*_*i*_’s changes. In all these cases, developers prefer to quickly bring the code back to its previous state to double check the implemented changes and understand the causes for the (possible) introduced issues.

In many cases we were not able to understand the reasons behind the reverted changes by manually inspecting the subject commits. These cases are just grouped in the root category *Reverted Commit*. Also, we observed that sometimes the code changes were reverted backward and forward within a few subsequent commits.

Our study is not the first one investigating reverted commits in software repositories. Shimagaki et al. (2016) conducted a study to gain a better understanding of why commits are reverted in large software systems. They found that 1%-5% of the commits from the systems they studies are reverted and this number could be reduced by improving team communication and developers’ awareness. However, in some cases, commits are reverted due to external factors (e.g., requirement change by end-users, customers, or remote teams) and, in this case, they are difficult to avoid. Yan et al. (2019) proposed a model to automatically identify commits that will be reverted in the future. They also found that the developer who performs the change is the most important predictive feature among the three they studied (i.e., code change, developer, commit message).  Besides the recommendations to developers already provided by Shimagaki et al. (2016),  the presence of reverted commits in the history of software systems is also relevant for the mining software repositories (MSR) research community. For example, it could be debated whether studies analyzing the change-proneness of code components (i.e., how frequently code components are subject to changes in software repositories) — e.g., Bieman et al. (2003), Catolino and Ferrucci (2019), and Aniche et al. (2018) — should take into account commits that are quickly reverted or, as currently done, should consider them. The same applies for works using the history of changes implemented by developers as a proxy for the developers’ experience — e.g., Rahman et al. (2017) and Tufano et al. (2017). In Section 3 we present an empirical study aimed at assessing the impact of considering (or not) reverted commits for typical MSR data collection tasks.

#### Documentation (49)

Our last category groups remedy commits related to software documentation. These commits impact a number of documentation artifacts that represent the main subcategories (see Fig. 4), namely: release notes, licensing statements, code comments, commit messages, and readme files.

The errors fixed in release notes, licenses and readme files are mostly minor. For example, some commits just update the copyright year in a previously committed file. Also, the fixes of commit messages rarely happen, and are mostly due to adding a missing commit message for the code changes implemented in the previous commit.  Also these cases are interesting for the MSR community. For example, approaches using pairs 〈*code changes implemented in a commit* *c*_*x*_, *commit message of* *c*_*x*_〉 to train models able to learn how to generate commit notes (Jiang et al. 2017), could be negatively biased by commit messages in a commit *c*_*i*+ 1_ referring to changes implemented in *c*_*i*_.

Other remedy commits are related to code comments. In some cases, developers documented the rationale for a code change implemented in the previous commit. This is the case of commit (Commit to jitsi project on GitHub 2021a) performed in the project. In a commit (Commit to jitsi project on GitHub 2021b) they fix a bug due to the wrong generation of a message where they mistakenly set a value of a parameter to an empty string instead of a value.

In the next commit (Commit to jitsi project on GitHub 2021a) they add a comment to explain the otherwise non-trivial difference in the generated message.

Interesting is also the missed removal of Self Admitted Technical Debt (SATD) instances (Potdar and Shihab 2014), meaning technical debt documented by developers in the code with comments such as $\mathtt {TODO: \dots }$, $\mathtt {TOFIX: \dots }$, etc. We found cases in which developers payed-back the technical debt instance, but forgot to remove the comment documenting the SATD. This resulted in a code-comment inconsistency (Wen et al. 2019), that could possibly confuse developers comprehending the associated code components. One representative example of this scenario is the commit (Commit to tinkerpop project on GitHub 2021a) performed in the project where the developers “*Forgot to remove todo from previous commit*”, as their commit message says. Indeed, in the remedy commit they remove a single-line comment which says “*todo: need a test to enforce this condition*”, and just right in the previous commit (Commit to tinkerpop project on GitHub 2021b) they had implemented the missing test case, thus paying back the technical debt.

 The cases discussed above for the *Documentation* category provide us with some interesting lessons learned. First, identifying code components in which specific types of comments (e.g., to document the rationale for a given implementation and/or to detail the application logic) are needed, can be a promising research direction. Second, automatically classify SATD as payed-back (or not) can help in identifying obsolete and misleading comments in the code. We believe this is another interesting research direction for the software engineering community.

## Study II: On the Impact of Reverted Commits on MSR Data Collection

### Study Design

The *goal* of the study is to investigate the impact of reverted commits (one subcategory of quick remedy commits) on data collection activities performed in the context of MSR studies. The *purpose* is to show the level of noise introduced by reverted commits in MSR studies collecting specific types of data. Our study addresses the following research question:

**RQ**_**2**_: *What is the impact of reverted commits on data collection tasks when mining Java projects?*

We instantiate RQ_2_ on two popular “data collection tasks”, namely the identification of bug-fixing commits (Rodriguez-Perez et al. 2017b; Rodríguez-Pérez et al. 2018; Tufano et al. 2018; Wang et al. 2020; Penta et al. 2020) and of refactoring operations (Penta et al. 2020; Peruma 2019; Mahmoudi et al. 2019; Lin et al. 2019; Fakhoury et al. 2019; AlOmar et al. 2019) performed in the change history of software systems. We show the impact of filtering-out (or not) reverted commits while mining this data (e.g., a refactoring operation mined in the system’s history in commit *c*_*i*_ may have been reverted in commit *c*_*i*+ 1_, thus questioning its validity as a study data point). The results of our study help to increase the awareness about noisy data points introduced by reverted commits, eventually leading to a better handling of data processing in MSR studies.

#### Data Collection and Analysis

To answer RQ_2_, we sorted the 1,497 projects used in the context of RQ_1_ based on the number of commits in their change history impacting at least one source code (i.e., Java) file. We discarded seven projects having more than 100k of such commits since the data extraction process for refactoring operations on these systems is too costly in terms of time. In particular, we run the data collection process described in the following for two weeks, processing in parallel up to ten systems at a time. At the end of these two weeks, the seven systems we excluded were still far from being processed. We replaced these seven systems with those ranked in positions 101-107, still selecting a total of 100 repositories as context for RQ_2_. The list of considered projects is available in our replication package (Replication package 2021).

From each of the 100 selected projects we extracted the following information:

- **Bug-fixing commits.** To identify bug-fixing commits in open-source repositories, we mined lexical patterns in commits, as done in previous work (Fischer et al. 2003). In particular, we used the pattern defined by Tufano et al. (2019), who reported a precision of 97.6% (i.e., 97.6% of commits identified by this heuristic as bug-fixes are true positives): The commit message must match the patterns *(“fix” or “solve”) and (“bug” or “issue” or “problem” or “error”)* to classify the related commit as a bug-fix.
- **Refactoring operations.** To mine the refactoring operations in the history of a system at commit level we used the state-of-the-art tool RefactoringMiner (Tsantalis et al. 2018; Tsantalis et al. 2020). If at least one refactoring operation is identified in a given commit, we mark this commit as a “refactoring commit” and store the refactoring-related information (i.e., performed refactoring operations, code lines impacted by the refactoring).
- **Reverted commits.** Before detailing the procedure we adopted to identify reverted commits, it is important to clarify that, in our study, we only focus on identifying commits reverting Java code changes from the previous commit. This means that, as for our previous study, we are still in a scenario in which we are looking at pairs of commits *c*_*i*_ and *c*_*i*+ 1_, with *c*_*i*_ being the reverted commit and *c*_*i*+ 1_ the reverting one. We implemented an approach similar to the one by Yan et al. (2019). First, we identify reverting commits by scanning commit messages, looking for the pattern *reverts commit* *c*_*i*_. Second, to identify reverting commits *c*_*i*+ 1_ not explicitly labeled as such in their commit note, we compare the code they change with the one changed in the previous commit *c*_*i*_. To do this, we stored the changes performed in each commit in a vector having the format: 〈*AddedFile, DeletedFile, ModifiedFile, AddedCode, DeletedCode*〉. We refer to this 5-element vector as a commit change vector *V*, in which *AddedFile* indicates the added file paths, *DeletedFile* the deleted file paths, *ModifiedFile* the modified file paths, and *AddedCode* and *DeletedCode* refer to the text in the inserted lines and removed lines, respectively, with each line added together with a prefix of the changed file path. Given the commits *c*_*i*_ and *c*_*i*+ 1_, we mark *c*_*i*_ as a reverted commit and *c*_*i*+ 1_ as a reverting commit if they satisfy all of the following constraints:
  - *addedFile*_*i*+ 1_ = *deletedFile*_*i*_,
  - *deletedFile*_*i*+ 1_ = *addedFile*_*i*_,
  - *modifiedFile*_*i*+ 1_ = *modifiedFile*_*i*_,
  - *addedCode*_*i*+ 1_ = *deletedCode*_*i*_,
  - *deletedCode*_*i*+ 1_ = *addedCode*_*i*_.
- **Partially reverted commits.** Similarly to the identification of completely reverted commits, given two commits *c*_*i*_ and *c*_*i*+ 1_, we mark *c*_*i*_ as a partially reverted commit and *c*_*i*+ 1_ as a partially reverting commit if they satisfy all of the following constraints:
  - *addedFile*_*i*+ 1_ ⊂*deletedFile*_*i*_,
  - *deletedFile*_*i*+ 1_ ⊂*addedFile*_*i*_,
  - *modifiedFile*_*i*+ 1_ ⊂*modifiedFile*_*i*_
  - *addedCode*_*i*+ 1_ ⊂*deletedCode*_*i*_,
  - *deletedCode*_*i*+ 1_ ⊂*addedCode*_*i*_.

Once extracted the above described data from the change history of the 100 selected projects, we compute the impact of considering/not-considering completely and partially reverted commits when collecting bug-fixes and refactoring operations from the change history of software projects. In particular, given a task *T* ∈{*refactorings*, *bugfixes*}, we compute for each project the average number of noisy data points introduced by a single reverted commit in the following way:

$$ \frac{|DataPoints_{T_{all}} - DataPoints_{T_{cleaned}}|}{|reverted|} $$

where $DataPoints_{T_{all}}$ represents the total number of data points collected for the task *T* (i.e., in our case, number of bug-fixes or number of refactorings); $DataPoints_{T_{cleaned}}$ is the number of data points collected for the same task *T* when removing reverted commits; and |*reverted*| is the total number of reverted commits identified in the repository. To make an example, in the case of *T* = mining of bug-fixing commits, a value for this metric of 0.5 indicates that every reverted commit introduces in the collected data, on average, 0.5 noisy bug-fixing commits. We compute the same metric when considering both reverted and partially reverted commits:

$$ \frac{|DataPoints_{T_{all}} - DataPoints_{T_{cleaned'}}|}{|reverted| + |partially reverted|} $$

In this case, the only difference is that $DataPoints_{T_{cleaned'}}$ represents the number of data points collected for the task *T* when removing both reverted and partially reverted commits.

### Results

We start by commenting on the number of fully and partially reverted commits we identified in the 100 systems. Overall, we found 5,083 reverted (avg= 51, median= 30, Q1 = 15, Q3 = 60) and 958 partially reverted (avg= 10, median= 7, Q1 = 3, Q3 = 13) commits. While the number of reverted commits is non-negligible, we only found a limited number of partially reverted commits, with a maximum of 44 observed for apache/hbase. Also, fully reverted commits are found in all repositories, while for the partially reverted ones we did not find any instance in six of the analyzed projects. Note that the number of reverted commits found in our paper is substantially lower as compared to the data reported in the work by Shimagaki et al. (2016) and Yan et al. (2019), in which up to 5% of commits in a repo were found to be reverted. However, it is worth noting that in our study, differently from previous work, we only considered reverting commits *c*_*i*+ 1_ that revert changes in *c*_*i*_ (e.g., we do not consider *c*_*i*+ 1_ as reverting commit if it reverts changes performed in *c*_*i*− 1_).

**Fig. 5:**
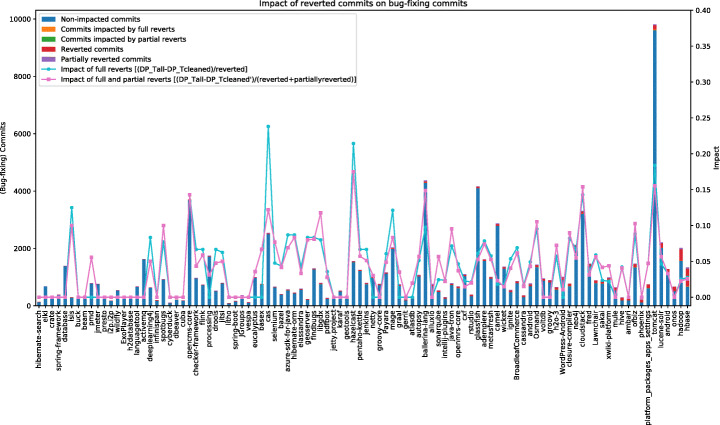
Impact of reverted commits on bug-fixing commits

Figure 5 shows the results achieved for the data collection task related to bug-fixing commits. The 100 projects are sorted from the left to the right in ascending order by the absolute number of completely reverted commits. For example, the first project on the left is hibernate/hibernate-search with only one reverted commit in its change history, while the last is apache/hbase with 617. The stacked bar chart shows the number of non-impacted bug-fixing commits (i.e., commits that are not fully nor partially reverted)—blue bar, of fully reverted bug-fixing commits (orange bar) and of partially reverted bug-fixing commits (green bar), using the scale on the left *y*-axis. The partially reverted commits are hardly visible in the chart due to their low number.

The line chart in Fig. 5 shows instead the average impact of fully reverted commits (cyan line) and of both fully and partially reverted commits (pink line) using the formulas presented at the end of Section 3.1. In this case, the reference *y*-axis is the one on the right. Since the number of partially reverted commits is very low, we limit our discussion to the impact of fully reverted commits on the collected bug-fixes. However, as it can be seen in the line chart in Fig. 5, the trend of the two lines is very similar.

Ignoring reverted commits from the data collection has an impact, in terms of collected data points, on 57 out of the 100 analyzed systems. The average impact goes from a minimum of 0.02 (i.e., a reverted commit results, on average, in 0.017 “wrong” bug fixes collected) to a maximum of 0.24, with an average of 0.07 and a median of 0.06. The system resulting in the highest number of noisy data points for this task is apache/tomcat, in which the 147 reverted commits cause the collection of 27 reverted bug-fixes (on average, each reverted commit contributes 0.18 noisy data points).

We discuss a few examples of commits that were identified as a bug-fixing commit but had been reverted in the subsequent commit.

One commit of the project was marked bug-fixing https://github.com/apache/hadoop/commit/cb64e8eb192 as the log message said: “*Fix synchronization issues …*” The changes, however, were reverted by the next commit with the message “ *Revert* “*Fix synchronization issues* $\dots $” *because forgot to add JIRA Number*.” In this case, the reverted commit is indeed a bug-fixing commit, but the reverting commit should not be considered a valid bug-fix even though it contains the expression “Fix issues.” In the worst case, a mining study might believe that there are already two bug-fixes in the change history after the revert. While in reality, the code does not implement the bug-fix after the revert.

In another commit of the project https://github.com/apache/tomcat/commit/f711963768, the author claimed in the commit message that a reported issue had been fixed. However, the fix was reverted in the subsequent commit as they noticed that “*it fixes the reported issue but introduces other issues*.” Again, the fix was reverted, and the reverting commit should not be counted as a bug-fix.

Another interesting example can be seen in https://github.com/aosp-mirror/platform_packages_apps_settings/commit/d3dcce029d. The bug-fix was reverted because the issue had been fixed before by someone else: “*Revert […] Bug: 27700406” Framework bug was fixed by ag/900274, so this is no longer needed.*”

It is important to highlight that, while there is an impact of the reverted commits on the collected bug-fixes (and, as such, excluding them from the data analysis might be preferred), such an impact is overall limited. However, it is also worth reminding that in our study design we favored the precision in the identification of reverted commits rather than recall. Thus, the number of reverted commits we identify is certainly an underestimation of the real ones. Also, in case these reverted bug-fixes are used to compute additional data (e.g., are provided as input to an SZZ algorithm as done in previous works (Penta et al. 2020)), such an error can further propagate and results in additional noisy data points. Basically, a cleaning of reverted commits when collecting bug-fixes is usually desirable, even though for specific study designs (e.g., collection of bug-fixing commits for qualitative manual analysis) it might not be needed.

**Fig. 6:**
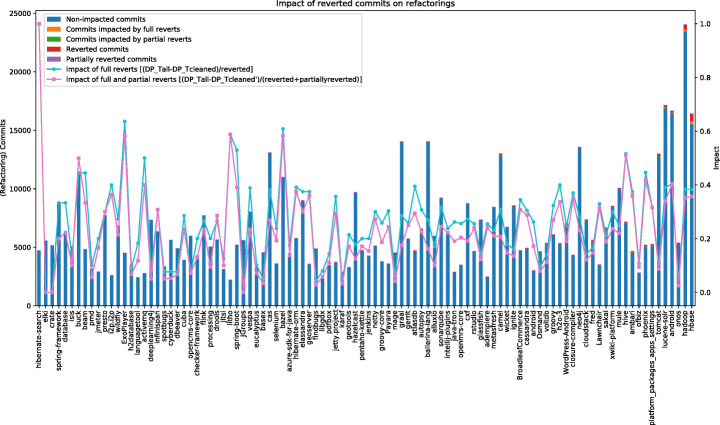
Impact of reverted commits on refactoring commits

Figure 6 shows the same data discussed before for the refactoring-related task, with the only difference that, in this case, the reverted and partially reverted commits are “refactoring commits”, meaning commits featuring at least one refactoring operation. Also in this case we focus our discussion on the completely reverted commits.

Considering reverted commits during the data collection has an impact on 97 out of the 100 systems, with an average impact for a single reverted commit of 0.27 noisy data points (i.e., reverted refactoring commits), median= 0.26. The average impact goes from a minimum of 0.08 to a maximum of 1.00. The latter is a sort of outlier, since it refers to the hibernate/hibernate-search that, as said before, does only have one reverted commit that is indeed a refactoring commit.

In this case, the system that would be mostly affected by the presence of noisy refactoring commits collected when not handling reverted commits is apache/hbase with a total of 236 reverted refactoring commits that would be wrongly considered (result of the overall 617 reverted commits in this system).

An example of refactoring-related commits that have been reverted is the one commit performed in the project https://github.com/metasfresh/metasfresh/commit/7875c81632. The developer performed some refactoring operations (e.g., rename parameter, change return type, rename method), but the commit message claimed that the refactoring was only partially. The subsequent commit reverted this partial refactoring. Thus, specific types of empirical studies mining refactoring operations may consider ignoring the refactorings detected in the first commit, since the refactorings were implemented and quickly reverted by the developer.

In another commit performed in the project,^2^ one of the private inner classes has been moved to a public outer class through a move class refactoring. However, the author said that this refactoring was only for testing purpose and reverted the change in the subsequent commit.

As compared to the collection of bug-fix commits, reverted commits seem to have a higher impact when mining refactoring operations, with an overall of 1,447 reverted (noisy) refactoring commits that are identified across the 100 analyzed systems. Considering our conservative approach to identify reverted commits, we believe its cleaning is highly recommended when studying refactoring operations over the history of software systems.

### Summing Up

Both the quantitative and qualitative results of this study point to an opportunity to obtain cleaner data by considering reverted commits: Reverted commits are noise in the recorded history of a system, and while it looks like a negligible phenomenon, we argue that the cleaner the data the better the analyses. In the spirit of the work by Kawrykow and Robillard (2011) on cleaning out non-essential changes from any mining software repositories research, detecting and removing reverted commits could thus also become a part of the cleaning preprocessing before starting an actual analysis.

## Threats to Validity

Threats to *construct validity* concern the relation between the theory and the observation, and in this work are mainly due to (Study I) the manual analysis we performed to identify the reasons behind the quick remedy changes performed by developers, and (Study II) the heuristics used to identify bug-fixing commits and reverted commits as well as to imprecisions introduced by the tool used to mine refactoring operations.

To mitigate subjectivity bias in the manual analysis (Study I), every commit was assigned to two authors who manually analyzed it independently. Then, in the case of disagreement, a third author was assigned to the commit to solve the conflict. In addition to that, we used lexical patterns to identify candidate remedy commits. While these lexical patterns can return false positives, these have been excluded in our study through manual validation, and thus do not influence our findings.

Concerning Study II, the identification of bug-fixing commits was based on a heuristic defined and validated in previous work (Tufano et al. 2019). As for the reverted commits, we combined two types of heuristics based on the analysis of the commit message and of the code changes. Also, we limited the identification of reverted commits only to pairs of subsequent commits to increase the precision in our analysis. While this likely reduces the number of reverted commits we can identify (i.e., recall), considering the analysis we performed (i.e., assessing the average “cost” in terms of noisy data of a single reverted commit) our findings should not be substantially affected. Finally, refactoring operations have been mined by relying on the state-of-the-art tool RefactoringMiner (Tsantalis et al. 2020).

Threats to *internal validity* concern external factors we did not consider that could affect the variables and the relations being investigated. One aspect could be related to the selection of projects being considered. As explained by Kalliamvakou et al. (2014) mining GitHub can be risky because projects may contain very few commits. To mitigate this threat, we applied strict criteria (i.e., more than 500 commits, more than ten stars) when selecting the context of our study. Also, we manually looked into the set of retrieved projects to exclude repositories that do not represent real software systems (e.g., tutorials, collections of code examples) and forked projects. Also, in Study I all considered data points (i.e., commits) have been manually checked, strengthening its internal validity.

Threats to *external validity* concern the generalizability of our findings. Our analysis in Study I is limited to a specific set of 500 commits we randomly selected as the output of a keyword-based mechanism used for the pre-selection of commits likely to be “remedy” commits. Because of this procedure, our taxonomy inevitably omits types of remedy commits we did not analyze and/or documented in diverse data sources. Also, we set a 5-minute threshold to identify the *quick remedy commits* subject of our study. While our choice is justified by the temporal distribution plotted in Fig. 2, changing this threshold value may result in different findings. This investigation is part of our future research agenda.

As for Study II, the reported findings are related to a set of 100 Java open source projects, which do not allow us to generalize our results to projects written in other languages which require additional investigations.

## Related Work

There is a vast literature of empirical studies investigating developers’ commits for various purposes. Many studies tackle research questions related to when, where, or why developers change source code. However, there has been little research on quick fixes, or consecutive changes performed by software developers, as well as on the impact of specific types of commits in the data collection of MSR studies. Here we present an overview of the related work close to the topic of this article.

### Reasons for Changes

Mockus and Votta (2000) studied a large legacy telecommunication system to identify reasons for software changes. Using an automatic classification algorithm, they discovered three primary reasons for changes according to maintenance activities: adding new functionality (*adaptive*), repairing faults (*corrective*), and restructuring the code to accommodate future changes (*perfective*). They noticed that several changes fall under the fourth category of inspection rework changes, i.e., changes to implement the recommendations of code inspections. They also found a strong relationship between the type and size of a change and the difficulty of a change.

Hattori and Lanza (2008) conducted an empirical study on nine large open source systems. They defined the size of a commit based on the number of files. They classified commits according to the comments information into development or maintenance (reengineering, corrective engineering, and management).

Hindle et al. (2008) conducted a study on large commits, created a taxonomy of their purpose. They found that large commits are more focused on perfective maintenance, while small commits are more related to corrective maintenance.

### Effects of a Change on Quality

#### Small Changes

Purushothaman and Perry (2005) investigated small source code changes (i.e., one-line changes) during the development process. An interesting finding of their work is that there is less than a four percent probability that a one-line change introduces a fault in the code.

#### Large Changes

Śliwerski et al. (2005) studied fix-inducing changes, i.e., changes that lead to problems indicated by fixes. In particular, they investigated the day of the week and the size of commits in Eclipse and Mozilla. They found that the commits performed on Friday and large commits have higher chances of introducing bugs.

#### Social Characteristics

Eyolfson et al. (2011) investigated the bug-fix time as the time from the earliest commit that introduced the bug to the bug-fixing commit. Their findings suggest that the time and date of a code update may affect the quality of the code.

In an earlier study, Claes et al. (2018) also studied developers’ working hours by investigating the timestamps of commit activities. They found that developers mainly work in regular office hours, and they did not find support that project maturation would decrease irregular working hours.

Bird et al. (2011) mined commits in Windows Vista and Windows 7 to investigate the relationship between code ownership and software quality. They found that high levels of ownership, specifically high values for the proportion of ownership for the top owners, or high values for major, and low values of minor contributors, are associated with fewer defects.

Rahman and Devanbu (2011) found that implicated code is more closely related to the contribution of a single developer. Their findings also indicate that an author’s specialized experience in the target file is more important than general experience.

Gonzalez-Barahona et al. (2011) investigated in FLOSS projects from the Mozilla community whether contributors fixing a bug are the same introducing and seeding them in the first place. Their results show that in 80% of the cases, the bug-fixing activity involves source code modified by at most two developers. Hence, in most of the cases, the bug fixing process is not carried out by the same developers.

#### Supplementary Patches

Park et al. (2012) studied bugs whose initial patches were later considered incomplete and to which programmers applied supplementary patches. They examined three open source projects: Eclipse JDT core, Eclipse SWT, and Mozilla. They found that a significant portion of bugs fall in this category while their causes are often diverse, e.g., missed port changes, incorrect handling of conditional statements, or incomplete refactoring. In their follow-up work (Park et al. 2014; 2017) they further investigated supplementary patches, and the results showed that only 7% to 17% of supplementary patches had content similar to their initial patches, which implies that a separate code clone analysis could not predict the supplementary patch location.

An et al. (2014) found that supplementary bug fixes accounted for 10.3% to 26.9% of total bug reports. Also, in the subject systems, a high percentage of the supplementary fixes (i.e., from 21.6% to 33.8%) had been re-opened.

#### Consecutive Changes

Dai et al. (2014) investigated the relationship between consecutive changes and software quality. They studied two concepts of consecutive changes: chain of consecutive bug-fixing file versions, and chain of consecutive file versions where each pair of adjacent versions has different authors. They found that those consecutive changes have a negative impact on the later file versions in the short term, especially when the length of the change chain is four or five.

#### Inconsistent Changes

Bettenburg et al. (2012) conducted an empirical study on inconsistent changes to code clones in two large open source software systems. They observed that the number of defects caused by inconsistent changes to code clones was substantially lower at the release level, compared to the revision level. Their findings suggest that developers can effectively manage and control the evolution of cloned code at the release level.

#### Incorrect Changes

Yin et al. (2011) presented a comprehensive characteristic study on incorrect bug-fixes from large operating system code bases, including Linux, OpenSolaris, and FreeBSD. They found that at least 14.8%-24.4% of sampled fixes for post-release bugs in these large operating systems were incorrect.

#### Changes and Refactoring

Palomba et al. (2017) conducted a quantitative investigation of the relationship between different types of code changes and different refactoring types. They found that developers tend to apply a higher number of refactoring operations when they are fixing bugs.

Bavota et al. (2012) presented a study aimed at investigating to what extent refactoring activities induce faults. They showed that refactorings involving hierarchies (e.g., *pull down method*) induce faults very frequently. Conversely, other kinds of refactorings are likely to be harmless in practice.

### Changes and Time

Rodriguez-Perez et al. (2017a) conducted two case studies and studied the *Time To Notify* (TNN) metric which describes how much time it takes for a bug to be notified/reported since the bug was introduced into the source code. They examined how this metric is related to software maintenance and evolution. Interestingly, they found relatively high mean values of TTN in the projects: 312 and 431 days.

Kim and Whitehead (2006) studied the bug-fix time of files in ArgoUML and PostgreSQL. Their statistics showed that fixing 50% of the bugs requires 100 to 300 days, while the median bug-fix time is about 200 days.

### Change Patterns

Pan et al. (2009) presented an automatic approach in which software history data is mined to find patterns in bug fix changes and automatically categorize bugs. They defined bug fix patterns (e.g., method call with different actual parameter values) which covered 45-63% of bug fixes in seven open source projects.

Zhao et al. (2017) conducted an empirical study to investigate the characteristics of change types in bug fixing code. They proposed a change classification schema and developed an automatic classification tool to categorize changes into five change types. They found that interface-related code changes are the most frequent bug-fixing changes.

In a related research thread, Martinez and Monperrus (2019) presented Coming, a tool to mine change pattern instances from git commits.

Change patterns have also been exploited recently to train neural networks in order to automatically reproduce code changes implemented by developers in pull requests of open source projects (Tufano et al. 2019) or to learn how to automatically fix bugs (Tufano et al. 2018).

### Bias and Noise in Mining Change Histories

Many approaches and studies depend on the quality of the dataset produced by mining change histories. Discussions about the bias in data collected by mining repositories have gained more attention recently. As Bird et al. (2009) say in a study on bias in bug-fix datasets: “*bias is a critical problem that threatens both the effectiveness of processes that rely on biased datasets to build prediction models and the generalizability of hypotheses tested on biased data*”. Here, we overview the potential causes and impact of bias and noise in mining studies.

#### Impact of Non-Essential Changes

Kawrykow and Robillard (2011) observed that software changes are often accompanied by non-essential modifications, such as local variable refactorings, or textual differences induced as part of a rename refactoring. They studied code changes in over 24,000 changesets of seven open-source systems and observed non-essential changes in their history. They found that up to 15.5% of a system’s method updates were due to non-essential differences among interesting observations.

The authors also investigated the impact of non-essential changes on change-based analyses in their same research work (Kawrykow and Robillard 2011). They implement a method-pair association rule mining analysis similar to the approach of Zimmermann et al. (2004). This approach, given a set of changes, suggests and predicts likely further changes. They found that removing non-essential method updates improved the precision of the recommendations by 10.5% and decreased their recall by 4.2%.

#### Impact of Tangled Changes

Herzig and Zeller (2013) defined a tangle change as a single commit which consists of separate changes (e.g., fixing a bug and adding a new feature). They found that up to 15% of all bug fixes include tangled changes.

Later, they also showed that tangled changes could significantly impact the accuracy of defect prediction models assessed in empirical studies (Herzig et al. 2016).

#### Impact of Untracked Changes

Hora et al. (2018) claimed that changes affecting code entities’ names (untracked changes) present a potential threat to MSR studies. For example, a method rename could be misinterpreted as the deletion and the addition of a method, thus, splitting its history. Based on an empirical analysis of 15 Java systems, they found that between 10 and 21% of the method level changes are untracked, hence, should be systematically considered by MSR studies.

#### Bias in Bug Localization and Prediction

Kochhar et al. studied biases in bug localization (Kochhar et al. 2014). They identified potential causes that can impact the validity of the results reported in studies. One of the main reasons is that files modified in commits that fix the bugs might not contain the bug. Instead, files are often changed because of refactorings or modifications to program comments.

Kim et al. (2011) measured the impact of noise on defect prediction models built using historical defect data obtained by mining software repositories. They consider false positives and false negatives as noise in such dataset. They found that, for large defect datasets, noises alone do not lead to substantial performance differences. However, their prediction performance decreased significantly when the dataset contained 20%-35% of both FP and FN noises.

Rahman et al. (2013) assessed whether the size of the dataset or bias affects the performance of defect prediction approaches. Similar to the findings of Kim et al. (2011), they conclude that size matters at least as much as bias.

#### Noise in History Slicing

Li et al. (2016) presented a semantic history slicing approach to extract changes related to a particular functionality. As they say, state-of-the-art techniques tend to over-approximate the inferred changes, and their slice histories may contain irrelevant changes. Their approach implements a method to untangle unrelated changes introduced in a single commit.

#### Threats in Aggregating Software Repository Data

Robillard et al. (2018) investigated potential threats to validity associated with metrics that summarize software repository data. They conducted a case study in which they retrieved and analyzed every file considered abandoned to investigate the files’ properties, including size, file type, and amount of comments. As a result, they identified eight major threats that can generalize to software process metrics derived from repository data. These threats are fragility, file content, file role, comment, contributor involvement, quantization, architectural sensitivity, and exceptional action.

### Summing Up

As discussed above, previous work investigated code changes from several different points of view. However, to the best of our knowledge, our study is the first to investigate the impact of reverted commits on data collected by mining the versioning system (and, in particular, big-fixing commits and refactoring operations).

## Conclusion

**Fig. 7:**
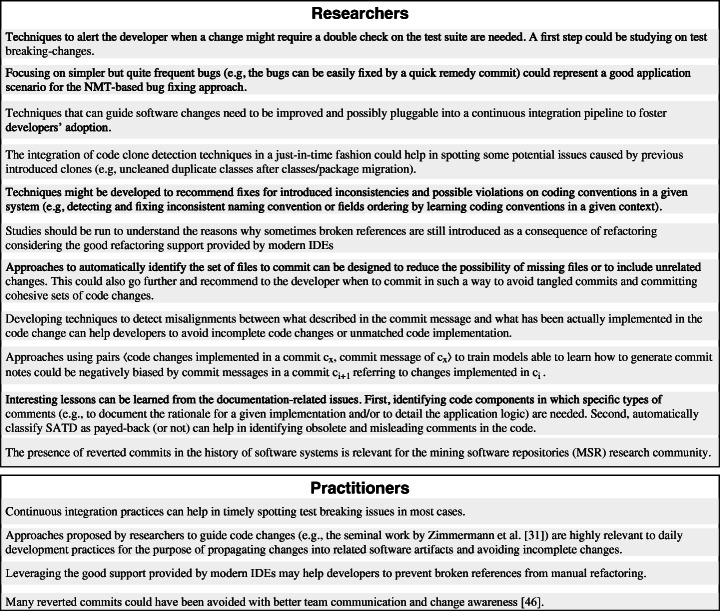
Summary implications for researchers and practitioners

We presented two empirical studies related to *quick remedy commits*. In the first, we qualitatively investigate *quick remedy commits* performed by developers in GitHub projects. We defined *quick remedy commits* as commits performed by developers to remedy changes omitted or errors introduced in a previous commit, performed just a few minutes before. This study (Study I) is based on the manual analysis of 500 commits, that we classified by looking at the objective of the remedy commit. The output of this study is represented by the taxonomy depicted in Fig. 4. We used several qualitative findings to distill lessons learned resulting in actionable items for both researchers and practitioners, which are summarized in Fig. 7.

Then, we investigated the impact of a specific type of quick remedy commits, namely reverted commits, on the data extracted for MSR studies. In particular, we focused on two data collection tasks performed in many previous works: (i) the identification of bug-fixing commits and (ii) the mining of refactoring operations over the change history of a system. Our analysis disclosed the amount of potential noise brought by reverted commits for these two data collection tasks.

### Future Work

Our future work will target two directions. First, we will work on some of the research directions discussed in the results section of Study I, and summarized in the following:

#### Automatic bug fixing

Developing approaches able to learn how to automatically fix the “simple” bugs that, as shown in our study, are fixed by developers within a few minutes from their introduction. We believe that approaches based on deep learning (see e.g., Tufano et al. 2018) can be particularly performant in this specific context.

#### Automatic identification of omitted changes

Integrating approaches to identify locations for missed code changes in a continuous integration pipeline, to alert developers when changes they are committing are likely to be incomplete.

#### Learning coding conventions

Investigating novel techniques to learn coding conventions, enlarging the set of conventions that are currently supported by state-of-the-art techniques (Allamanis et al. 2014). Once learned, the coding conventions can be automatically checked on the code to commit, raising a warning in case violations are detected.

#### Automatic software documentation

Developing techniques able to (i) identify code components in which specific types of comments (e.g., rationale for implementation choices) are needed; and (ii) automatically classify SATD as payed-back (or not).

Second, we will analyze the impact of other types of *quick remedy commits* on the outcome of MSR studies. For example, the results of works mining logical coupling between components (i.e., how often specific files co-change), can be impacted by considering a *quick remedy commitc*_*i*+ 1_ as part of its previous commit *c*_*i*_, since they basically represent the same implementation activity.

## Footnotes

1 Our online appendix features an analysis of common keywords present in the commit message of these commits in comparison to non-quick-remedy commits (Replication package 2021).

2 https://github.com/wordpress-mobile/WordPress-Android/commit/c363f2ff2d
